# Transmission dynamics of COVID-19 among index case family clusters in Beijing, China

**DOI:** 10.1017/S0950268820002848

**Published:** 2020-01-19

**Authors:** Ying Cao, Yueh Wang, Aritra Das, Calvin Q. Pan, Wen Xie

**Affiliations:** 1 Center of Liver Diseases, Beijing Ditan Hospital, Capital Medical University, Beijing, China; 2 Biostatistics, Data Sciences, Safety & Regulatory, Research & Development Solutions, IQVIA, Taibei, Taiwan; 3 Epidemiology and Outcomes Research, Real World Solutions, IQVIA, Haryana, India

**Keywords:** Basic reproductive number, coronavirus disease 2019, family clusters, severe acute respiratory syndrome coronavirus 2, transmission dynamics

## Abstract

The outbreak of coronavirus disease-2019 (COVID-19) impacts public health dramatically around the world. The demographic characteristics, exposure history, dates of illness onset and dates of confirmed diagnosis were collected from the data of 24 family clusters from Beijing. The characteristics of the cases and the estimated key epidemiologic time-to-event distributions were described. The basic reproductive number (*R*_0_) was calculated. Among 89 confirmed COVID-19 patients from 24 family clusters, the median age was 38.0 years and 43.8% were male. The median of incubation period was 5.08 days (95% confidence interval (CI) 4.17–6.21). The median of serial interval was 6.00 days (95% CI 5.00–7.00). The basic reproductive number (*R*_0_) was 2.06 (95% CI 2.02–2.08). The median of onset-to-care-seeking days and the median of onset-to-hospital admission days were significantly reduced after 23 January 2020, which implied the enhanced public health awareness among families. With epidemic containment measures in place, the results can inform health authorities about possible extent of epidemic transmission within families. Furthermore, following initiation of interventions, public health measures are not only important for curbing the epidemic spread at the community level but also improve health seeking behaviour at the individual level.

## Introduction

Since the second half of December 2019, a cluster of cases with ‘pneumonia of unknown aetiology’, potentially linked to a live animal market, started getting reported from the city of Wuhan in China which has since then spread to rest of China and countries across the globe [1]. The causative organism for these atypical pneumonia cases was subsequently identified as a novel coronavirus (2019-nCoV), later rechristened to severe acute respiratory syndrome coronavirus 2 (SARS-CoV-2), which has been found to be different from two recent epidemic causing coronaviruses – MERS-CoV and SARS-CoV [2]. This makes it the seventh identified member of the coronavirus family that can act as human pathogen. The corresponding disease has been called coronavirus disease 2019 (COVID-19).

The COVID-19 outbreak has become perhaps the greatest public health emergency in recent history, having given rise to more than 1.3 million confirmed cases and almost 80 000 deaths globally until 8 April 2020 [3]. Of these, nearly 82 000 cases and 3335 deaths have been reported from mainland China [4]. Proper understanding of the epidemiologic parameters of an infectious disease (such as reproduction numbers, incubation period, etc.) is an essential criterion for designing public health interventions. As the disease was first described in China, several studies have already described the epidemiologic parameters of COVID-19 disease in China, especially based on data from early stages of the epidemic [5–9]. Some heterogeneity was noted in the parameters reported by the cited studies. For example, the value of *R*_0_ or the expected number of secondary cases generated by an index case in a fully susceptible population, reported by the aforementioned studies varied between 2.2 [6] and 3.8 [10]. This is not entirely unexpected as different populations may have different proportions of susceptible (or immune) individuals that may affect *R*_0_. Also, the value of *R*_0_ can evolve with epidemic progression owing to different environmental and biological factors [11]. Despite the heterogeneity of findings, these studies shared one characteristic – all of them utilised data from Wuhan/Hubei province, which has been the epicentre of COVID-19 outbreak in China. Given the demographic and climatic diversity in China, the characteristics of the epidemic reported from Wuhan may not be applicable all across the country. Furthermore, different mutations of SARS-COV-2 have been identified from different countries/regions and pathogenicity has been found to vary according to the type of mutation [12], which also highlight the importance of describing the epidemiologic parameters at the regional level and, in turn, for public health decision-making. Against this backdrop, we utilised data from index case family clusters in Beijing to describe the several epidemiologic parameters including serial interval, incubation period, growth rate (*r*), epidemic doubling time (*T*_d_), *R*_0_, etc.

## Methods

### Data source

The study dataset comprised of 120 individuals belonging to 24 families (clusters) who were hospitalised/quarantined in Beijing Ditan Hospital, affiliated to Capital Medical University in Beijing, which is a hospital designated for diagnosis and treatment of COVID-19 patients. The families were selected from line-list of hospital attendees based on following eligibility criteria: (1) presenting to the aforementioned hospital for either treatment or quarantine; (2) at least two family members diagnosed with COVID-19 and (3) date of COVID-19 diagnosis (availability of positive result) between 15 January 2020 and 14 February 2020. Besides diagnostic data (test results and corresponding dates), the following information were collected through interviews with 24 index patients and their family members: demographic information, time of illness onset and date(s) and type(s) of healthcare seeking related to current illness (both OPD visits and hospitalisation). A confirmed case of COVID-19 was defined as having at least two positive results for SARS-CoV-2 by real-time reverse transcription polymerase chain reaction (RT-PCR) assays, regardless of the clinical signs and symptoms of COVID-19 disease. The index case for each family cluster was defined as the RT-PCR positive family member with earliest exposure history or (in the absence of such history) presenting with symptoms before any other family members. The maximum follow-up period for detecting the emergence of secondary cases was 14 days. However, it was reported that the incubation period of COVID-19 can extend beyond 14 days [13]. Therefore, in order to be conservative, the RT-PCR negative family members were followed for up to 45 days to ensure that there were no false-negatives. Among 120 individuals, there were 65 RT-PCR-positive secondary cases, 24 index cases and 31 RT-PCR negative cases.

### Statistical analysis

The analysis focused on estimating the following epidemiologic parameters: serial interval, *r*, *T*_d_, *R*_0_, onset-to-care-seeking interval and onset-to-hospital admission interval.

*R*_0_ was calculated using the exponential growth methodology described by Wallinga and Lipsitch [14]. This method assumes that the number of infected subjects grows exponentially with *r*. This method also requires estimation of serial intervals or the time period between infection of an index case and a secondary case generated from the index case. We calculated serial interval using the following formula:
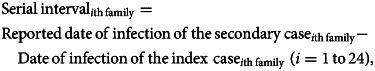
where for the secondary cases, the date of infection was defined as either the date of testing for the first RT-PCR positive result or the date of symptom onset, whichever is earlier. Published studies on COVID-19 epidemic in China [10, 13] have reported the incubation period to be between 2 and 14 days. Therefore, to maintain biological plausibility, if the value of serial interval for any family turned out to be lower than 2 days, then that value was excluded from the analysis of serial interval.

Among 65 serial intervals, an empirical distribution consisting of 49 serial intervals was obtained by the above steps (16 serial intervals with serial intervals less than 2 days were excluded). The median of serial interval distribution and corresponding 95% bootstrapped confidence interval (CI) (obtained from 10 000 replicates) were calculated.

**Fig. 1.**
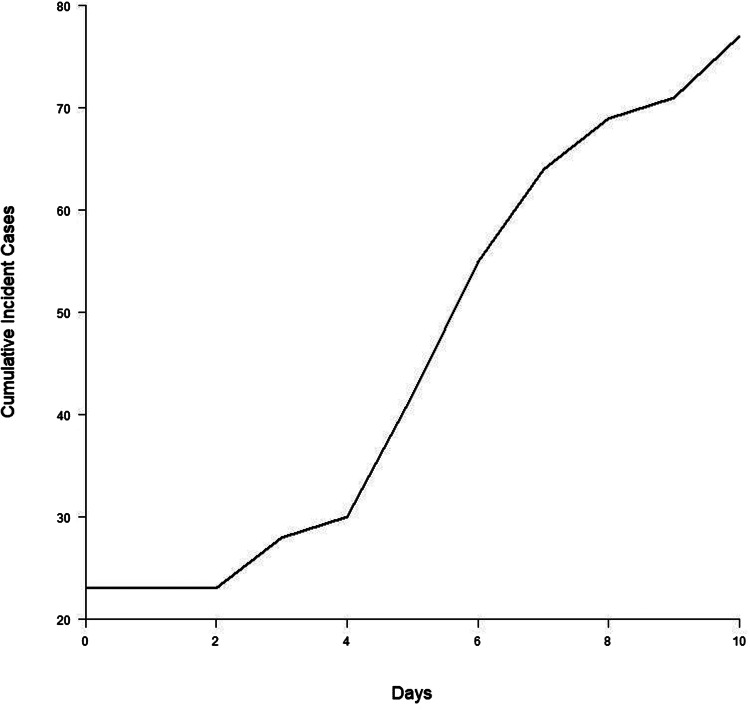
Cumulative incident cases by 10 days since first infection.

Furthermore, we assumed that the number of infected cases followed an exponential growth at a fixed growth rate. To mimic the exponential growth phase (assumed to be up to 10 days from the diagnosed date of index case), we transformed the obtained serial intervals in 10 days to cumulative incident cases, as shown in Figure 1, to estimate *r* using the least squares method [15]. Note that the family clusters were close population, the increasing trend of incident case number would be slow down, hence no exponential growth anymore if we included incidence data longer than 10 days. The following equation was used for the estimation of *r*:
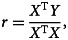
where superscript T denotes matrix transposition, *X* = {1, 2, …, 10}^T^ is the vector of exponential growth period, recorded in days:
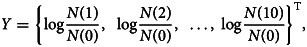
where *N*(*t*) denotes the cumulative case number at *t*th day since the onset of infection, and *N*(0) is the index case number.

*T*_d_ was estimated from *r* using the following equation:
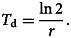


Finally, *R*_0_ was calculated using the following equation:
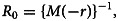
where *M* is a moment generating function of the serial interval. We assumed empirical distribution of serial interval to derive *M*. The 95% CIs of *R*_0_, *r* and *T*_d_ were also using bootstrap with 10 000 replicates.

### Incubation period

We calculated the incubation period using the simple mid-point imputation method described by Cai *et al*. [16]. Using this approach, the incubation period for each secondary case was obtained by the following formula:
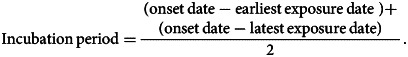


Furthermore, to obtain the median and 95% CI, we modelled incubation period assuming a log-normal distribution. As before, 95% CI of median was obtained using bootstrap.

### Onset-to-care-seeking interval and onset-to-hospital admission interval

The onset-to-care-seeking and onset-to-hospital admission intervals were modelled assuming Weibull distribution and were stratified by date of illness onset before or after 23 January 2020 (the day of lockdown initiation). The median value with corresponding 95% CI (via bootstrap with 10 000 replicates) was calculated for both parameters. The null distributions for the test of difference between median for the cases having onset before 23 January and after were also obtained using bootstrap.

All analyses were conducted using R version 3.4.1 software.

## Results

The study population comprised of 24 index patients (one from each family cluster) and 96 family members. Among the family members, 65 were identified as secondary cases (which included 18 children below 15 years of age). The median age of the patients was 38.0 years (interquartile range (IQR) 29.0–58.0) and 39 (43.8%) of them were males. In total, 53 (59.6%) patients had recent travel history to Wuhan or reported having close contacts with individuals visiting from Wuhan. On stratifying the patients into pre- (illness onset before 23 January 2020) and post-lockdown periods, we found that the patients with pre-lockdown onset were significantly older, were more likely to be males and were more likely to report travelling to Wuhan or having contact with visitors from Wuhan compared to patients with post-lockdown onset.

**Fig. 2.**
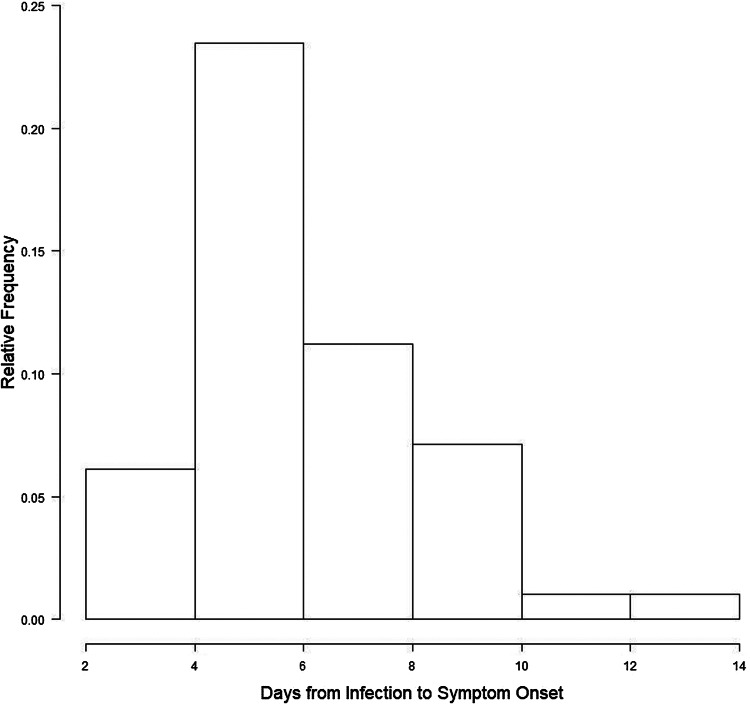
Serial interval empirical distribution.

The empirical distribution of serial interval is presented in Figure 2. The median serial interval was 6.00 (95% CI 5.00–7.00). Based on serial interval empirical distribution, the estimates (and 95% CI) of *R*_0_, *r* and *T*_d_ were 2.06 (2.02–2.08), 0.12 (0.11–0.12) and 6.00 days (5.59–6.60), respectively.

**Fig. 3.**
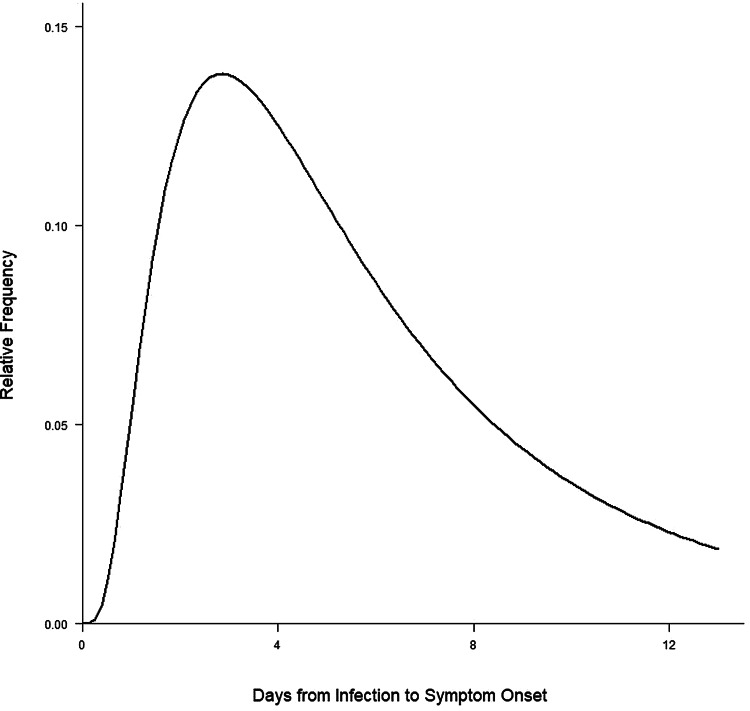
Incubation period distribution (fitted using log-normal distribution).

The distribution of incubation period (fitted using log-normal distribution) is presented in Figure 3. The median incubation period was 5.08 days (95% CI 4.17–6.21).

**Fig. 4.**
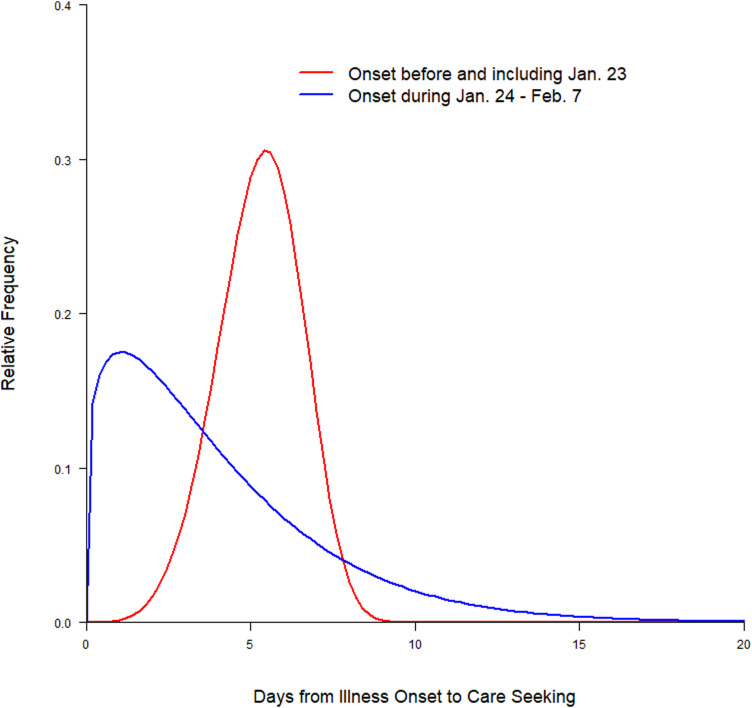
Distributions of onset-to-care-seeking interval (fitted using Weibull distribution).

**Fig. 5.**
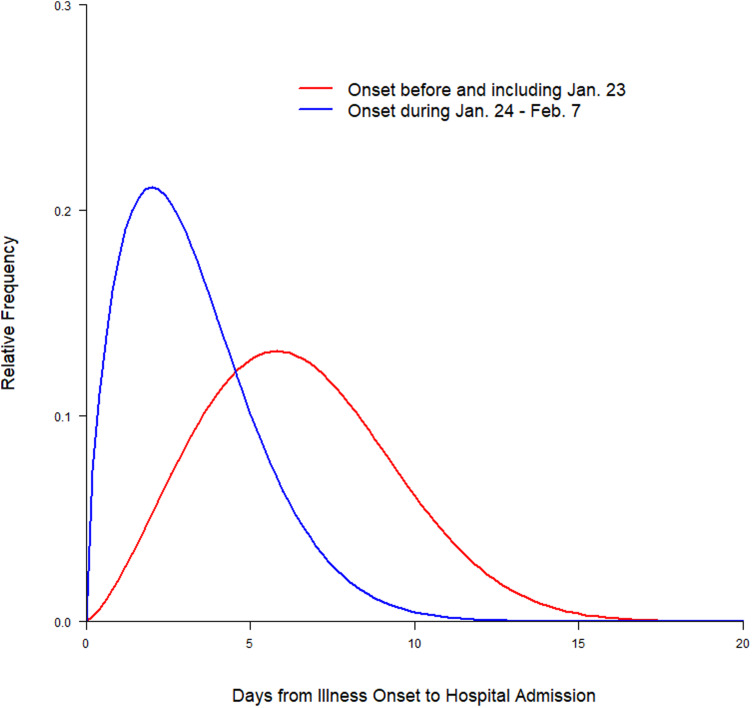
Distributions of onset-to-hospital admission interval (fitted using Weibull distribution).

The distributions of onset-to-care-seeking interval (fitted using Weibull distribution) in the pre- and post-lockdown periods are depicted in Figure 4. The median onset-to-care-seeking interval in the post-lockdown period was 3.16 days (95% CI 2.18–4.45), which was significantly shorter (*P* = 0.004) than those with illness onset in the pre-lockdown period (5.32 days; 95% CI 4.36–6.24). The Weibull distributions of onset-to-hospital admission interval for the pre- and post-lockdown periods are presented in Figure 5. As seen with onset-to-care-seeking, the median interval for onset-to-hospital admission was significantly shorter (*P* < 0.001) in the post-lockdown period (2.89 days; 95% CI 2.29–3.57) than that in the pre-lockdown period (6.28 days; 95% CI 4.40–8.41).

## Discussion

The current study used mathematical modelling to determine several epidemiologic parameters and transmission dynamics of COVID-19 outbreak from data on 24 family clusters in Beijing. We also compared demographic and care-seeking parameters between the cases with illness onset before and after the lockdown, possibly the most important public health intervention towards epidemic containment. The natural history of disease in this patient population will be published separately. To the best of our knowledge, this is one of the first attempt to assess the epidemiologic characteristic of the epidemic in China using a dataset that did not have any representation from Wuhan (Table 1).

**Table 1.** Demographics of family clusters with COVID-19 in Beijing as of 14 February 2020

Characteristics	Before 23 January (pre-lockdown)	24 January to 7 February (post-lockdown)
	(*n* = 20)	(*n* = 69)
Median age (IQR), years	54.5 (36.5 to 61.5)	36.0 (15.0 to 57.5)
Age range, years		
<15	1 (5.0%)	17 (24.6%)
15–44	6 (30.0%)	25 (36.2%)
45–64	10 (50.0%)	21 (30.4%)
⩾65	3 (15.0%)	6 (8.7%)
Male (%)	8 (40.0%)	31 (44.9%)
Exposure history		
Exposure to Wuhan	9 (45.0%)	17 (24.6%)
Exposure to person from Wuhan	4 (20.0%)	23 (33.3%)

Our data revealed that three out of five patients had either travelled to Wuhan or had contact with a visitor from Wuhan. However, the link with Wuhan was much more prominent among the patients having onset before lockdown (or the index cases) than among the patients who had post-lockdown onset, which suggests the presence of local human-to-human transmission in the later stage of the epidemic and supports implementation of interventions like social distancing for slowing the spread of infection. This corroborates with the findings from prior studies conducted in China and other countries [5, 16–18]. Children (<15 years old) accounted for a minor proportion of the infected in the pre-lockdown period. This could be because of higher vulnerability of infection among the elderly or could be attributed to the fact that children were more likely to be asymptomatic/mildly symptomatic and may not have been detected during the early stage of the epidemic [6]. However, in the post-lockdown period, children constituted approximately one-fifth of all patients. This corroborates with published reports [19]. Thus, we recommend that existing surveillance mechanism should also include children under its radar as the mild/asymptomatic children may act as potential source for human-to-human transmissions.

We detected an *R*_0_ of 2.06, indicating that each index case would give rise to approximately two secondary cases, if the surrounding population (family members in the context of current study) was completely susceptible. Given the narrow CI, we can conclude that the infection is likely to result in a sustained epidemic in Beijing (or at least in similar case family clusters), unless appropriate interventions are put in place. The *R*_0_ value is similar to that reported from a study conducted on COVID-19 outbreak in a cruise ship (*R*_0_ = 2.28) [20]. This is expected as, from the perspective of human-to-human transmission of infection, cruise ship passengers and family clusters are likely to have similar risk characteristics. Also, the *R*_0_ value is within the range of possible *R*_0_ (1.4–2.5) reported by the WHO [21] and other publications based on early stage epidemic in China [17]. Nevertheless, as the epidemic progresses, the *R*_0_ is likely to evolve as well and its value may need to be reassessed [11, 22]. The *R*_0_ estimated by us constitute an important component of the transmission dynamics of COVID-19 in Beijing, especially for local level transmissions (stage-II of an epidemic), and may serve as a critical reference point for epidemic control measures in Beijing. Furthermore, the incubation period and serial interval in this study were found to be slightly lower than that reported from Wuhan [6, 8]. Nonetheless, the estimates are quite close to Wuhan and the slight decrease may be attributed to the difference in study population between Wuhan-based studies (population-based) and the current study (index case family cluster).

The significant decrease noted in the intervals for onset-to-care-seeking and onset-to-hospital admission could be consequences of system-level (increased surveillance activities for case detection) as well as individual-level (increased public awareness about the disease and its symptoms) factors. This augur well for epidemic control measures as earlier care-seeking and hospitalisation suggests that infected patients, including asymptomatic individuals, are put on treatment/isolation more rapidly, which is likely to limit possible sources of infection.

The study had a few limitations. The inferences are drawn from a relatively small sample of family clusters. Although we admit that data scarcity may influence the precision of the results, the sample size was not much inferior from some other studies reporting on epidemiologic parameters of COVID-19 outbreak [6]. Furthermore, the sample size appears more acceptable given the scope of the study, which was to assess the parameters for the case family clusters rather than the entire susceptible population. Second, although we followed established methods for estimating the epidemiologic parameters, the results were dependent on several modelling assumptions including the assumptions about distributions. Additionally, it is possible that the time to diagnosis and hospitalisation may have been underestimated as these were estimated from the close contacts of index cases. However, given the increased awareness following the detection of the index case, the family members were expected to have COVID-19 tests quickly, if not immediately. Therefore, even if present, we consider that the extent of underestimation to be small. Finally, as with most research reporting on *R*_0_ of an infectious disease, the current study also assumed that the secondary cases arose from a completely susceptible population and the sources of infection for all secondary cases were respective index cases. These assumptions are unlikely to hold true in a real-world scenario.

## Conclusion

Notwithstanding the limitations, our study provides reliable estimates of several epidemiologic parameters of COVID-19 outbreak in index case family clusters in Beijing, China. Although several publications have reported on different epidemiologic parameters from China, the current study stands out for couple of reasons: (1) this is one of the first explorations of COVID-19 transmission dynamics from non-Wuhan data (collected from the national capital and largest city in China) and (2) the study data are derived from index case family clusters, which may mimic the post-lockdown scenario (as the families may be largely confined within their homes and practicing social distancing) better than population-based data. Despite its limited sample size, the findings of the current study will contribute to the existing body of evidence on COVID-19 outbreak and will help in devising appropriate public health strategies for the city of Beijing (and other Chinese metropolises).

## Data Availability

The data that support the findings of this study are available from the corresponding author on reasonable request. Participant data without names and identifiers may be shared with other researchers after approval from the corresponding author and the authorities including the Institutional Review Board and National Health Commission.

## References

[1] The 2019-nCoV Outbreak Joint Field Epidemiology Investigation Team QL (2020) An outbreak of NCIP (2019-nCoV) infection in China – Wuhan, Hubei Province, 2019–2020. China CDC Weekly 2, 79–80. PMC8393104 34594812

[2] Zhu N (2020) A novel coronavirus from patients with pneumonia in China, 2019. The New England Journal of Medicine 382, 727–733. 31978945 10.1056/NEJMoa2001017 PMC7092803

[3] World Health Organization (2020) Coronavirus disease 2019 (COVID-19) situation report–79. https://www.who.int/docs/default-source/coronaviruse/situation-reports/20200408-sitrep-79-covid-19.pdf?sfvrsn=4796b143_6 (Accessed 9 April 2020).

[4] National Health Commission of the People's Republic of China (2020) Update on COVID-19 as of 24:00 on April 8, 2020. http://www.nhc.gov.cn/xcs/yqtb/202004/fa7bb40a7fbf4b2c8f3989d512fe5b77.shtml (Accessed 9 April 2020).

[5] Chan JF (2020) A familial cluster of pneumonia associated with the 2019 novel coronavirus indicating person-to-person transmission: a study of a family cluster. Lancet (London, England) 395, 514–523. 10.1016/S0140-6736(20)30154-9 PMC7159286 31986261

[6] Li Q (2020) Early transmission dynamics in Wuhan, China, of novel coronavirus-infected pneumonia. The New England Journal of Medicine 382, 1199–1207. 31995857 10.1056/NEJMoa2001316 PMC7121484

[7] Sanche S (2020) High contagiousness and rapid spread of severe acute respiratory syndrome coronavirus 2. Emerging Infectious Diseases 26, 1470–1477. 32255761 10.3201/eid2607.200282 PMC7323562

[8] Zhang J (2020) Evolving epidemiology and transmission dynamics of coronavirus disease 2019 outside Hubei province, China: a descriptive and modelling study. The Lancet Infectious Diseases 20, 793–802. 32247326 10.1016/S1473-3099(20)30230-9 PMC7269887

[9] Zhao S (2020) Preliminary estimation of the basic reproduction number of novel coronavirus (2019-nCoV) in China, from 2019 to 2020: a data-driven analysis in the early phase of the outbreak. International Journal of Infectious Diseases 92, 214–217. 32007643 10.1016/j.ijid.2020.01.050 PMC7110798

[10] Linton NM (2020) Incubation period and other epidemiological characteristics of 2019 novel coronavirus infections with right truncation: a statistical analysis of publicly available case data. Journal of Clinical Medicine 9, 538. 10.3390/jcm9020538 PMC7074197 32079150

[11] Delamater PL (2019) Complexity of the basic reproduction number (*R*_0_). Emerging Infectious Diseases 25, 1–4. 10.3201/eid2501.171901 PMC6302597 30560777

[12] Yao H (2020) Patient-derived mutations impact pathogenicity of SARS-CoV-2. medRxiv. doi: 10.1101/2020.04.14.20060160.

[13] Lauer SA (2020) The incubation period of coronavirus disease 2019 (COVID-19) from publicly reported confirmed cases: estimation and application. Annals of Internal Medicine 172, 577–582. 32150748 10.7326/M20-0504 PMC7081172

[14] Wallinga J (2007) How generation intervals shape the relationship between growth rates and reproductive numbers. Proceedings of the Royal Society B-Biological Sciences 274, 599–604. 10.1098/rspb.2006.3754 PMC1766383 17476782

[15] Ma J (2020) Estimating epidemic exponential growth rate and basic reproduction number. Infectious Disease Modelling 5, 129–141. 31956741 10.1016/j.idm.2019.12.009 PMC6962332

[16] Cai QC (2006) Refined estimate of the incubation period of severe acute respiratory syndrome and related influencing factors. American Journal of Epidemiology 163, 211–216. 16339050 10.1093/aje/kwj034 PMC7109871

[17] Li C (2020) Asymptomatic and human-to-human transmission of SARS-CoV-2 in a 2-family cluster, Xuzhou, China. Emerging Infectious Diseases 26, 1626–1628. 32228809 10.3201/eid2607.200718 PMC7323514

[18] Rothe C (2020) Transmission of 2019-nCoV infection from an asymptomatic contact in Germany. The New England Journal of Medicine 382, 970–971. 32003551 10.1056/NEJMc2001468 PMC7120970

[19] Dong Y (2020) Epidemiology of COVID-19 among children in China. Pediatrics 145, e20200702. 32179660 10.1542/peds.2020-0702

[20] Zhang S (2020) Estimation of the reproductive number of novel coronavirus (COVID-19) and the probable outbreak size on the Diamond Princess cruise ship: a data-driven analysis. International Journal of Infectious Diseases 93, 201–204. 32097725 10.1016/j.ijid.2020.02.033 PMC7110591

[21] World Health Organization (2020) Laboratory testing for 2019 novel coronavirus (2019-nCoV) in suspected human cases. https://www.who.int/health-topics/coronavirus/laboratory-diagnostics-for-novel-coronavirus.

[22] Das A (2020) An approximation-based approach for periodic estimation of effective reproduction number: a tool for decision-making in the context of coronavirus disease 2019 (COVID-19) outbreak. Public Health 185, 199–201. 32653628 10.1016/j.puhe.2020.06.047 PMC7328541

